# Acute Beetroot Juice Supplementation Enhances Intermittent Running Performance but Does Not Reduce Oxygen Cost of Exercise among Recreational Adults

**DOI:** 10.3390/nu14142839

**Published:** 2022-07-11

**Authors:** Ozcan Esen, Raúl Domínguez, Raci Karayigit

**Affiliations:** 1 Department of Health Professions, Manchester Metropolitan University, Manchester M15 6GX, UK; 2 Institute of Sport, Manchester Metropolitan University, Manchester M155TN, UK; 3 Departamento de Motricidad Humana Rendimiento Deportivo, Universidad de Sevilla, 41013 Sevilla, Spain; rdherrera@us.es; 4 Faculty of Sport Sciences, Ankara University, 06830 Ankara, Turkey; rkarayigit@ankara.edu.tr

**Keywords:** nitric oxide, ergogenic aid, sports nutrition, beetroot juice, exercise performance

## Abstract

Nitrate (NO_3_^−^) supplementation has been reported to enhance intermittent exercise performance; however, its impact on oxygen (O_2_) cost during intermittent running exercise is unclear. The aim of this study was to assess if acute NO_3_^−^ supplementation would elicit performance benefits in recreationally active individuals during the Yo–Yo intermittent recovery level 1 (Yo-Yo IR1) test, with its potential benefit on O_2_ consumption (VO_2_), in a double-blind, randomized, crossover study, 12 recreational males consumed NO_3_^−^-rich (NIT; ~12.8 mmol), and NO_3_^−^-depleted (PLA; 0.04 mmol) concentrated beetroot juice 3 h before completing the Yo-Yo IR1 test. VO_2_ was measured at 160, 280 and 440 m (sub-maximal) and when the test was terminated (peak). Performance in the Yo–Yo IR1 was greater with NIT (990 ± 442.25 m) compared to PLA (870 ± 357.4 m, *p* = 0.007). The VO_2_ was not significantly different at 160 m (1.92 ± 0.99 vs. 2.1 ± 0.88 L·min^−1^), 280 m (2.62 ± 0.94 vs. 2.83 ± 0.94 L·min^−1^), 440 m (3.26 ± 1.04 vs. 3.46 ± 0.98 L·min^−1^) and peak (4.71 ± 1.01 vs. 4.92 ± 1.17 L·min^−1^) between NIT and PLA trials (all *p* > 0.05). The present study has indicated that acute supplementation of NO_3_^−^ enhanced intermittent running performance but had no effect on VO_2_ during the Yo–Yo IR1 test in recreational young adults.

## 1. Introduction

The ergogenic effect of dietary nitrate (NO_3_^−^) supplementation is attributed to its reduction of NO_3_^−^ to nitrite (NO_2_^−^) and, subsequently, nitric oxide (NO) [1]. This ingestion of NO_3_^−^-rich sources is known to increase plasma NO_2_^−^ and be beneficial for reducing the oxygen (O_2_) cost for a given workload [2,3,4,5], improving muscle contractile properties [6,7], and supporting fatigue resistance [8,9,10]. Interestingly, it has been shown that a vegetable source is more effective than NO_3_^−^ salts [11], taken as a supplement (e.g., concentrated beetroot juice). Furthermore, existing evidence also supports the notion that the reduction of NO_2_^−^ to NO is enhanced in intra-muscular hypoxic conditions such as that observed within the skeletal muscle during high-intensity activity [12,13].The potential benefits of NO_3_^−^ supplementation were also shown in muscle contractile properties (e.g., evoked contractile force), and these effects seem in preferentially type II compared to type I muscle fibers [14]. This enhancement in muscle contractility after NO_3_^−^ supplementation has been attributed to improved calcium handling and release [6,7] and improved skeletal muscle blood flow and vascular conductance during submaximal efforts [15]. As such, enhanced intermittent running performance in moderately- [16,17] and well-trained individuals [18] following NO_3_^−^ supplementation might be associated with the potential type II fibers’ specific impact of NO_3_^−^ [6,15] as type II fibers are predominantly recruited to satisfy the high muscle contraction demands during high-intensity intermittent exercise [19].

The Yo–Yo intermittent recovery level 1 test (Yo–Yo IR1) is well-established, ecologically valid and commonly used [20]. There are three previous studies that investigated the influence of NO_3_^−^ on the Yo–Yo IR1 test. Wylie et al. [16] reported an improvement in the Yo–Yo IR1 by 4.2% amongst moderately-trained team sport players following very large dose of NO_3_^−^ (29 mmol) over 36 h before the test. Subsequent studies observed similar enhancement (3.9% and 3.4%) in the Yo–Yo IR1 test after the ingestion of 6.4 mmol for 5 days in moderately-trained team sport players [17] and a 12.8 mmol/day for 6 days in highly trained soccer players, respectively [18]. Besides the heavy recruitment of type II fibers during a transition from low to high metabolic rate [21,22], intermittent running also leads to a high O_2_ demand relative to O_2_ delivery. Therefore, combined with the possible effects on type II fibers, the enhanced Yo–Yo IR1 performance in the previous studies might also link to the potential benefits of NO_3_^−^ supplementation on exercise efficiency by reducing the O_2_ cost [8]. Oxygen consumption (VO_2_) at a given velocity is an endurance performance referred as running economy [23], and it is assessed because it reflects the energy cost of running [24]. Whilst most studies have reported an enhanced economy using acute and/or multiple days of NO_3_^−^ supplementation during steady-state endurance exercises [25], it is presently unclear whether the enhanced economy effect of NO_3_^−^ supplementation that was observed during steady-state endurance exercises might also occur during an intermittent exercise as the Yo–Yo IR1 test. As such, further research is required to assess the effect of NO_3_^−^ supplementation on the response of VO_2_ during intermittent running in humans.

The previous studies above which reported enhanced intermittent running performance applied short-term supplementation periods [16,17,18], but previous meta-analyses reported that there is no difference between acute (e.g., 2–3 h pre-exercise) and chronic dosing regimens (e.g., 1–15 days) of NO_3_^−^ on endurance exercise [26,27]. In addition, more recent meta-analyses have revealed that the benefits of NO_3_^−^ supplementation in power output generally are apparent following acute supplementation [14,28]. Despite consistent effects of NO_3_^−^ supplementation on the Yo–Yo IR1 test after multiple-day supplementation, to date, its effect after acute supplementation is yet to be determined. This is important to evaluate, as it would be a more practical and applicable nutritional intervention approach for performance in team sports.

Therefore, the aim of this study was to assess if acute supplementation of NO_3_^−^ would elicit performance benefits in recreationally active individuals during the Yo–Yo IR1 test, with its potential benefit on VO_2_ consumption. It was hypothesized that NO_3_^−^ supplementation would enhance performance and reduce VO_2_ cost during the Yo–Yo IR1 test.

## 2. Materials and Methods

### 2.1. Participants

The sample size of this study was based on a priori calculation using G*Power software (version 3.1.9.4, Universität, Düsseldorf, Germany). In determining the minimal estimated sample, we have considered two key outcomes, namely total distance achieved and the difference in overall change of VO_2_. Considering total distance, a standardized mean difference of 1.03 was used based on the work of Nyakayiru et al. [18]. Considering the difference in change of VO_2_, a standardized mean difference of 0.93 was used based on the work of Bailey et al. [8]. In both instances, a *t*-test family was used with matched pairs, a power of 0.80, a two-tailed approach, and α was set at 0.05. The results from these estimations indicated that sample of 10–12 participants would be sufficient to detect a difference between NO_3_^−^ (NIT) and placebo (PLA) supplementation. Twelve recreationally active males (mean ± SD: age 27 ± 10 years, body mass 78.1 ± 11.8 kg, stature 180.5 ± 5.3 cm) were recruited for this study. All participants were involved in regular moderate-intensity exercise ~3 days per week and muscle-strengthening activities ~2 days per week. The participants were non-smokers, healthy, and did not use dietary supplements at the time of data collection. All participants were university students in the Sport Science department and were familiar with the Yo–Yo IR1 test. Ethics approval for this study was given by the Manchester Metropolitan University Research Ethics Committee (Reference no: 33132). All participants were informed of the nature and possible risks of the experimental procedures before providing written informed consent.

### 2.2. Experimental Design

The participants visited the testing facility on two separate occasions. Participants were assigned in a randomized, double-blind, placebo-controlled, crossover design to consume either a NO_3_^−^-rich (NIT) or a NO_3_^−^-depleted concentrated beetroot juice (PLA). The experimental trials were all carried out at the similar time of the day (±2 h). Mean and standard deviation of ambient temperature, humidity and pressure during the two trials were 17 ± 2.1 °C, 56.0 ± 4.3% and 1018 ± 2 mbar, respectively. A four- to six-day washout period separated the supplementation periods, as suggested by Wylie et al. [9]. Each participant was asked to record their dietary intake in the 24 h before the first experimental trial and replicate this in the 24 h before the subsequent trial. Participants were instructed to avoid strenuous exercise and the consumption of alcohol and caffeine for at least 24 h before each experimental trial. Participants avoided using antibacterial mouthwash throughout the duration of the study due to its prevention of the reduction of NO_3_^−^ to NO_2_^−^ in the oral cavity [29].

### 2.3. Supplementation Protocol

The participants received 140 mL of concentrated beetroot juice (~12.8 mmol of NO_3_^−^; Beet it, James White Drinks Ltd., Ipswich, UK) or NO_3_^−^-depleted beetroot juice (~0.04 mmol of NO_3_^−^) as PLA. This chosen dose was based on current recommendation for the optimal ergogenic effect of NO_3_^−^ supplementation (5–13 mmol of NO_3_^−^) [1,9,27]. Participants consumed 2 × 70 mL the supplement 3 h before the test to coincide with peak plasma [NO_2_^−^] [9].

### 2.4. Procedures

Upon arrival at the testing facility (an indoor fourth-generation artificial grass pitch), participants completed a standardized warm-up (10 min), ending with the first two shuttles of the Yo–Yo IR1 test in order to familiarize themselves with the audio and initial speeds. After 10 min of passive recovery, a resting capillary blood lactate (BLa) sample was taken from the pad of the index finger of the left hand using the Lactate Pro-2 (Lactate Pro analyser, Arkay, Kyoto, Japan). Immediately after the Yo-Yo IR1, a second capillary BLa sample was taken. 

Participants had an online gas analyzer fitter using a custom-made harness with the device positioned on their back. Pulmonary gas exchange was measured continuously using a Cosmed K5 (Cosmed, K5, Cosmed, Rome, Italy) with the system set for breath-by-breath analysis. The calibration of the K5 gas analyzer was performed before each test, according to the manufacturer’s instructions including a gas, volume, carbon dioxide (CO_2_) and breathing frequency. Breath-by-breath VO_2_, CO_2_ production (VCO_2_) and minute ventilation (VE) data from each test were linearly interpolated to provide second-by-second values. Subsequently, mean VO_2_, VCO_2_ and VE were assessed during each run and recovery period and averaged to provide the overall mean VO_2_, VCO_2_ and VE during the run and recovery periods for each stage of the Yo–Yo IR1 test. The mean sub-maximal values of 160, 280 and 440 m were based on those previously used [30]. Peak values for each variable were considered as the highest value achieved during the test. Previous literature has reported that the COSMED K5 had excellent reliability for VO_2_ (CV: 4.4%, CI: 3.2–6.7%, concordance correlation coefficient [CCC]: 0.95), VCO_2_ (CV: 6.2%, CI: 4.5–9.7%, CCC: 0.92) and VE (CV: 6.9%, CI: 4.9–10.7%, CCC: 0.89) during more than 2 h of continuous field tests [31].

The Yo–Yo IR1 test has been described elsewhere [20]. Briefly, the test consists of repeated 2 × 20 m runs, interspersed by a 10 s active recovery period, at progressively increasing speeds controlled by audio bleeps from a portable audio system. First four shuttles were at the speed of 10–13 km·h^−1^ (0–160 m), then three shuttles at 13.5 km·h^−1^ (200–280 m) and four shuttles at 14.0 km·h^−1^ (320–440 m); thereafter, the speed increased 0.5 km·h^−1^ every eight shuttles (i.e., 760, 1080, 1400 m, etc.). The final distance successfully covered was recorded after the second failed attempt to meet the start/finish line in the allocated time.

### 2.5. Statistical Analysis

All data were presented as means ± SD. Differences between NIT and PLA in distance covered during the Yo–Yo IR1 test, VO_2peak_, VCO_2peak_ and VE_peak_, RER_peak_ and BLa pre- and post-exercise test were analyzed using a paired samples *t*-test. Effect sizes (*d*) were calculated through Cohen’s *d* as: large *d* > 0.8, moderate *d* = 0.8 to 0.5, small *d* = 0.5 to 0.2, and trivial *d* < 0.2 [32]. Differences in VO_2_, VCO_2_, VE and RER at 160, 280 and 440 Im were determined using two-way repeated-measure ANOVA (supplement × distance). In addition, effect size was calculated as partial eta-squared (ŋ_p_^2^) varying small (<0.25), medium (0.26–0.63) and large (>0.63) [33]. All data were analyzed using SPSS 27.0 (IBM Corp., Armonk, NY, USA). Significance was determined at *p* < 0.05.

## 3. Results

**Figure 1:**
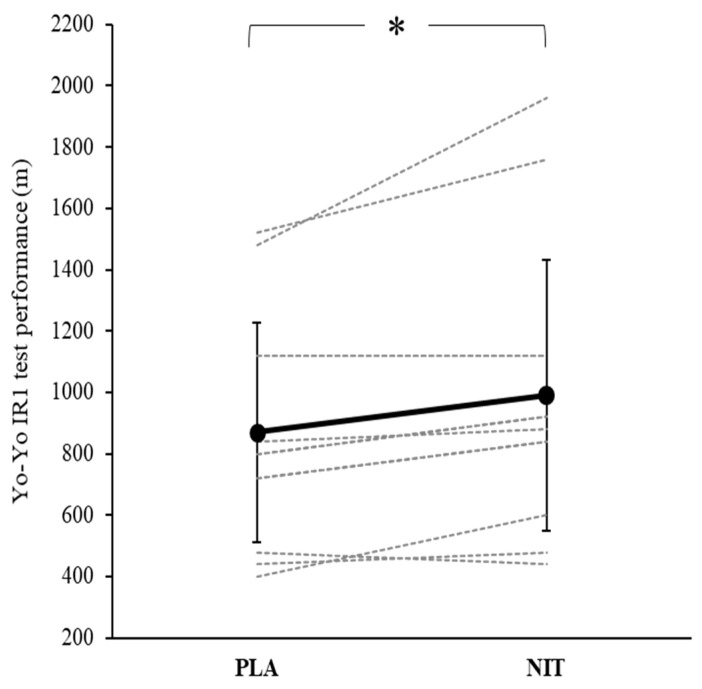
The distance covered in the Yo–Yo IR1 test was 14% greater with NIT compared to PLA. The dashed lines indicate the responses of individual participants. The solid line indicates the group mean (±SD). * *p* < 0.05.

The distance covered in the Yo–Yo IR1 test was significantly greater in NIT (990 ± 442.25 m) compared to PLA (870 ± 357.4 m, *p* = 0.007, *d* = 0.30, Figure 1).

**Figure 2:**
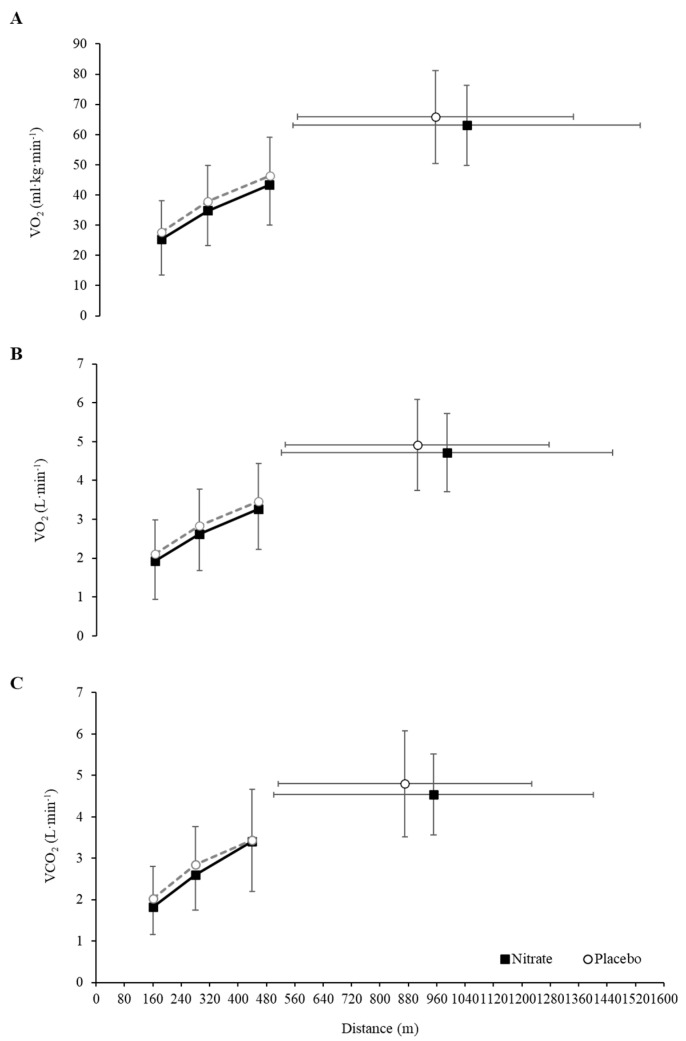
Relative (**A**) and absolute (**B**) pulmonary oxygen consumption (VO_2_), and absolute carbon dioxide production (VCO_2_) (**C**) responses during the Yo–Yo intermittent recovery level 1 test following dietary nitrate and placebo supplementation. Data are mean ± SD.

The group mean relative and absolute VO_2_ and absolute VCO_2_ responses at 160 m, 280 m, 440 m and peak during the Yo–Yo IR1 following both NIT and PLA supplementation were shown in Figure 2, and values (including VE and RER values) were reported in Table 1. The mean relative VO_2_ responses at submaximal distances were similar for NIT and PLA (ANOVA: supplementation, F = 2.27; *p* = 0.160; ŋ_p_^2^ = 0.171; supplementation × distance, F = 2.32, *p* = 0.131, ŋ_p_^2^ = 0.169), while these increased with distance (ANOVA: distance, F = 63.56; *p* < 0.001; ŋ_p_^2^ = 0.85). Similarly, the mean absolute VO_2_ responses at submaximal distances did not differ between NIT and PLA (ANOVA: supplementation, F = 1.14; *p* = 0.308; ŋ_p_^2^ = 0.094; supplementation × distance, F = 0.35, *p* = 0.966, ŋ_p_^2^ = 0.003), while these increased with distance (ANOVA: distance, F = 60.51; *p* < 0.001; ŋ_p_^2^ = 0.85). The mean VCO_2_ response were also very similar at submaximal distances between NIT and PLA (ANOVA: supplementation, *F* = 1.85; *p* = 0.201; ŋ_p_^2^ = 0.144; supplementation × distance, *F* = 1.84; *p* = 0.183; ŋ_p_^2^ = 0.143), while these increased with distance (ANOVA: distance, *F* = 40.26; *p* < 0.001; ŋ_p_^2^ = 0.785). There were also no significant differences between trials for the mean VE response (ANOVA: supplementation, *F* = 2.94; *p* = 0.114; ŋ_p_^2^ = 0.211: supplementation × distance, *F* = 2.12; *p* = 0.144; ŋ_p_^2^ = 0.161), while VE responses increased with distance (ANOVA: distance, *F* = 43.05; *p* < 0.001; ŋ_p_^2^ = 0.796). There were also no significant differences between conditions for RER (ANOVA: supplement, *F* = 0.46; *p* = 0.513; ŋ_p_^2^ = 0.040; distance, *F* = 0.34; *p* = 0.713; ŋ_p_^2^ = 0.030; supplementation × distance, *F* = 0.02; *p* = 0.946; ŋ_p_^2^ = 0.002).

There were no significant differences in the VO_2peak_ *p* = 0.114, *d* = 0.19), VCO_2peak_ (*p* = 0.085, *d* = 0.22), VE_peak_ (*p* = 0.295, *d* = 0.18), and RER_peak_ (*p* = 0.079, *d* = 0.80) between conditions. There were no significant differences in pre-BLa (*p* = 0.45, *d* = 0.36) and post-BLa (*p* = 0.24, *d* = 0.48) between NIT and PLA.

## 4. Discussion

This study set out to determine whether the acute supplementation of NO_3_^−^ would enhance the metabolic responses to intermittent running performance as measured via the Yo–Yo IR1 test among recreationally active adults. The original finding of the present study was that the acute supplementation of NO_3_^−^ via beetroot juice significantly enhanced intermittent running performance in the Yo–Yo IR1 test (by 14%, *p* = 0.007). However, the metabolic responses (e.g., VO_2_, VCO_2_ and VE) did not alter at submaximal distances (at 160, 280 and 440 m) or peak during the Yo–Yo IR1 test after acute NO_3_^−^ supplementation compared to placebo. These findings indicate that acute NO_3_^−^ supplementation can enhance performance without altering exercise efficiency during intermittent running. 

The present study shows that distance covered in the Yo–Yo IR1 test increased by 14% following the acute supplementation of NO_3_^−^. This finding is line with three previous studies that reported an enhanced performance in the Yo–Yo IR1 test by 4.2%, 3.9% and 3.4% after a large NO_3_^−^ dose (29 mmol) over 36 h before testing [16], a moderate NO_3_^−^ dose (6.4 mmol) for 5 days [17] and a high NO_3_^−^ dose (12.8 mmol) for 6 days [18], respectively. Our finding extends the findings of those previous studies and suggests that a similar performance benefit can be gained by an acute high dose of NO_3_^−^ supplementation (~12.8 mmol of NO_3_^−^ 3 h pre-exercise). It is interesting to see some of the best participants in the present study showed some of the greatest improvements. Whilst these individual greatest improvements might be an explanation of the much greater improvement in the Yo–Yo IR1 test in the present study compared to previous observations, this also supports the existence of potential responders and non-responders to NO_3_^−^ supplementation [1,34]. Marked differences exist between individuals in the erogenicity of NO_3_^−^ supplementation. Assuming the potential type II preference of NO_3_^−^ is on those factors, it might be speculated that because some of the participants in the present study had a high proportion of type II muscle fibers, this might have theoretically increased the ergogenic potential of NO_3_^−^ [34]. The greater improvement in the present study compared to the previous observations would be also due to a different population being used (recreational individuals vs. moderately/highly trained team-sport players) given that existing evidence has shown that potential ergogenic effect of NO_3_^−^ supplementation is more evident in individuals who have low levels of aerobic fitness [1,10]. Highly endurance-trained athletes could be less responsive to NO_3_^−^ supplementation mediated by a lower fraction of type II muscle fibers or other factors such as greater NO synthase activity, mitochondrial efficiency or better muscle oxygenation compared to moderately trained subjects [35]. Therefore, further research is required to determine whether acute (2–3 h pre-exercise) high-dose supplementation of NO_3_^−^ (>6 mmol) can benefit in highly trained team sports athletes. Given the considerable differences in technical requirements when executing specific skills, the metabolic cost of the activity and the participants’ characteristics, extrapolating these findings to every team sport activity and/or players is fraught. Future studies are therefore warranted to explore whether the findings of the present study can be reproduced in different team sports activities and/or athletes.

To best of our knowledge, this is the first study that assessed pulmonary VO_2_, VCO_2_ and VE responses following NO_3_^−^ supplementation during the Yo–Yo IR1 test. This study shows that NO_3_^−^ supplementation had no effect onVO_2_ response during the Yo–Yo IR1 test. These findings are consistent with observations that have reported an enhancement in intermittent exercise performance, consisting of repeated sprints, but no alteration in VO_2_ [16,36]. However, more recent studies reported inconsistent findings regarding the impact of NO_3_^−^ supplementation to improve performance or/and metabolic responses during different high-intensity intermittent exercise [37,38]. Whilst Kent at al. [37] found that supplementation of NO_3_^−^ did not enhance repeated-sprints performance in hypoxia, but may reduce VO_2_, Sousa et al. [38] showed no impact of NO_3_^−^ supplementation either on VO_2_ or repeated-sprint training. Besides inter-study differences (e.g., participant training status, supplementation regimen and environmental conditions), previous studies applied considerably different exercise modalities and exercise protocols regarding work-to-rest ratio (e.g., intensities, durations, and numbers of work and/or rest) [17,36,37,38,39]. In earlier studies, reduced VO_2_ following NO_3_^−^ supplementation during submaximal exercise [2,8,9,25] was attributed to a reduced adenosine-tri-phosphate (ATP) cost of muscle force production. The lack of effect in VO_2_ in this study and in previous studies conducted intermittent-exercise protocols might be, at least partly, due to the regular fluctuations in exercise intensity (e.g., non-steady-state conditions). Therefore, the findings of the present study suggest that the observed ergogenic effect of NO_3_^−^ in intermittent exercise may work through divergent mechanisms of action independent of alterations in the efficiency of oxidative metabolism.

Some other mechanistic underpinnings, such as improved muscle contractility, for the effect of NO_3_^−^ supplementation have been also identified [1]. Animal-based studies reported that the effect of NO_3_^−^ supplementation is more apparent in type II compared with type I fibers regarding physiological response and performance, such as improving force output selectively in type II fibers via increasing sarcoplasmic reticulum calcium handling and/or release [6,7]. Despite remaining to be elucidated in human, this mechanism may better explain the ergogenic basis for the enhanced performance in the Yo–Yo IR1 test where a greater recruitment of type II fibers is expected [19]. Improved performance in the Yo–Yo IR1 might be related to delaying fatigue as a result of preserving the reduction in muscle excitability [40,41]. Wylie et al. [9] have previously attributed enhanced performance in the Yo–Yo IR1 test to attenuated muscle excitability due to a net loss of potassium. Further, it has been reported that NO_3_^−^ supplementation attenuated the increase in motor unit action potential (MUP) duration [42]. Shorter MUP duration may result in a faster muscle fiber conduction velocity and greater sarcoplasmic reticulum calcium release and thus maintained force production in the face of fatigue development [43,44]. Taken together, these potential impacts of NO_3_^−^ supplementation in neuromuscular function and motor unit activity in combination of the potential type II preference of NO_3_^−^ might account, at least partly, for the enhanced intermittent running performance in the present study. However, it has yet to be fully understood what specific mechanism may underline ergogenic potential of NO_3_^−^ supplementation, and therefore, it is important to understand that these proposed mechanisms would work independently or in combination to contribute to enhance intermittent exercise performance following dietary NO_3_^−^ supplementation.

The ergogenic effect of NO_3_^−^ supplementation has been attributed to its capacity to elevate plasma NO_2_^−^ level with the subsequent reduction of circulating NO_2_^−^ to NO [1]. Although plasma NO_2_^−^ was not assessed after ~12.8 mmol of NO_3_^−^ supplementation in the present study, doses of 6.4–16.8 mmol of NO_3_^−^ have been consistently shown to enhance plasma NO_2_^−^ by a magnitude that would be expected to enhance exercise performance [1,28]. Indeed, acute supplementation with similar and/or lower doses of NO_3_^−^ has previously shown to enhance performance [9,45] and/or reduce VO_2_ [9,10,46,47]. The present study aimed consciously not to interfere in dietary intake behavior to accomplish lifestyle adequate exercise conditions, particularly as the dose of supplemented NO_3_^−^ was much higher than its intake in regular diet. Although we did not standardize the participants’ diets, they were instructed to recorded and reproduce their dietary intake first and subsequent trials in order to exclude a possible effect of dietary intake on performance on trial days. Considering BLa as an indicator of the anaerobic metabolism [48], the results of this study suggest the absence of effect of NO_3_^−^ supplementation in the anaerobic glycolytic. However, this study has reported a trend to statistical differences in RER_peak_ with a large effect size (*p* = 0.079, *d* = 0.80) with a higher contribution of carbohydrate to metabolism at the end of the Yo–Yo IR1 test. Based on the analysis of the effect sizes of an increased RER_peak_ within changes in BLa suggest a possible increase in the oxidative (and not anaerobic) metabolism of glucose at high intensity. This could be explained by hemodynamic and metabolic functions of NO as increased blow flow to muscles or an enhanced glucose uptake in the active muscles [49]. In fact, a higher utilization of oxidation of glucose during high intensity efforts could partially explain the enhancement in the time-to-exhaustion during high-intensity exercises [39,50]. Future studies should analyze the effect of the metabolic contributions of NO_3_^−^ supplementation during high-intensity efforts.

## 5. Conclusions

The present study has shown, for the first time, that acute high-dose supplementation of NO_3_^−^ enhanced the Yo–Yo IR1 test performance in recreational young adults. However, NO_3_^−^ supplementation had no effect on VO_2_ responses during the intermittent running, suggesting that the observed performance enhancement is likely related to other potential physiological influence of NO_3_^−^ (e.g., its type II fiber-specific effect and potential impact on neuromuscular functions), but not to its potential effect on O_2_ cost. These findings suggest that acute supplementation with NO_3_^−^-rich beetroot juice may be a nutritional ergogenic aid during intermittent running in recreational adults, and that further mechanistic research is required.

## Figures and Tables

**Table 1:** Submaximal and peak pulmonary gas responses during the Yo–Yo IR1 test, and pre- and post-exercise BLa.

	NIT	PLA
**VO_2_** (mL·kg·min^−1^)		
160 m	24.43 ± 11.92	27.75 ± 10.43
280 m	34.78 ± 11.43	37.80 ± 12.00
440 m	43.37 ± 13.26	46.34 ± 12.81
Peak	63.07 ± 13.21	65.87 ± 15.40
**VO_2_** (L·min^−1^)		
160 m	1.92 ± 0.99	2.10 ± 0.88
280 m	2.62 ± 0.94	2.83 ± 0.94
440 m	3.26 ± 1.04	3.46 ± 0.98
Peak	4.71 ± 1.01	4.92 ± 1.17
**VCO_2_** (L·min^−1^)		
160 m	1.82 ± 0.66	2.03 ± 0.78
280 m	2.61 ± 0.85	2.85 ± 0.92
440 m	3.42 ± 1.21	3.44 ± 1.23
Peak	4.54 ± 0.98	4.80 ± 1.28
**VE** (L·min^−1^)		
160 m	46.65 ± 22.17	55.21 ± 26.04
280 m	71.11 ± 25.91	79.93 ± 28.42
440 m	87.69 ± 32.48	89.39 ± 34.68
Peak	138.01 ± 25.59	143.27 ± 31.10
**RER** (VCO_2_/VO_2_)		
160 m	1.03 ± 0.19	1.01 ± 0.15
280 m	1.04 ± 0.13	1.02 ± 0.07
440 m	1.01 ± 0.15	0.98 ± 0.12
Peak	1.06 ± 0.23	0.98 ± 0.10
**Pre-BLa** (mmol·L^−1^)	2.1 ± 0.6	1.9 ± 0.5
**Post-BLa** (mmol·L^−1^)	12.8 ± 1.6	13.7 ± 2.1

## Data Availability

The data presented in this study are available on request from the corresponding author. The data are not publicly available due to restrictions privacy.
